# QUALITATIVE INSIGHT ON PROFESSIONAL EXPERIENCES WORKING WITH DIVERSE OLDER ADULT POPULATIONS

**DOI:** 10.1093/geroni/igae098.0317

**Published:** 2024-12-31

**Authors:** Rakshitha Mohankumar, Kristopher Kern, Brenna Renn

**Affiliations:** University of Nevada Las Vegas, Las Vegas, Nevada, United States; VA North Las Vegas Health Care System, North Las Vegas, Nevada, United States; University of Nevada Las Vegas, Las Vegas, Nevada, United States

## Abstract

The older adult population in the United States is expanding not only in number but also in diversity. Historically marginalized older adults face persistent and pervasive health disparities and barriers, spanning social, structural, and clinical factors, hindering access to services to help them age successfully and in a dignified manner. Regarding clinician barriers, training in the principles and practices of providing culturally respectful care is inconsistent. We sought to better understand ways that professional geropsychologists and trainees address diverse and intersectional identities during clinical practice and research with older adults. An online survey was distributed to the American Psychological Association Society of Clinical Geropsychology listserv. Questions included open-ended prompts asking about the ways in which respondents consider and engage in culturally responsive care to older adults with intersectional identities and those who have been historically marginalized by society, which were coded using qualitative thematic analysis. Respondents (n=28) were majority (81%) professional geropsychologists and primarily worked in the Department of Veterans Affairs, private practice, and/or academic settings. A common theme emerged of implementing a client-centered approach when working with this population, including adjusting existing measures and interventions to the needs of the individual and engaging in anti-discriminatory practices. Common limitations included desire for concrete strategies to increase access to care, working with LBTQIA+ older adults, and reducing stigma for seeking services. These results provide qualitative insight into how such issues are conceptualized in clinical practice across the U.S. and point to opportunities for training and professional development.

